# Contemporary women prisoners health experiences, unique prison health care needs and health care outcomes in sub Saharan Africa: a scoping review of extant literature.

**DOI:** 10.1186/s12914-018-0170-6

**Published:** 2018-08-06

**Authors:** Marie Claire Van Hout, Rosemary Mhlanga-Gunda

**Affiliations:** 1 Public Health Institute, Liverpool John Moore’s University, L32ET, Liverpool, UK; 2 College of Health Sciences, Centre for Evaluation of Public Health Interventions, Department of Community Medicine, University of Zimbabwe, Harare, Zimbabwe

**Keywords:** Sub Saharan Africa, Prisons, Women, Human immunodeficiency virus infection, HIV

## Abstract

**Background:**

Sub Saharan African (SSA) prisons have seen a substantial increase in women prisoners in recent years. Despite this increase, women prisoners constitute a minority in male dominated prison environments, and their special health needs are often neglected. Research activity on prison health remains scant in SSA, with gathering of strategic information generally restricted to infectious diseases (human immunodeficiency virus infection HIV/tuberculosis TB), and particularly focused on male prisoners. Health care provisions for women (and pregnant women) in SSA prisons are anecdotally reported to fall far short of the equivalence care standards mandated by human rights and international recommendations, and the recent agreements set out in the Southern African Development Community (SADC) Minimum Standards for HIV in Prisons.

**Methods:**

A scoping review mapped what is currently known about women prisoners’ health experiences, unique prison health care needs and health care outcomes in SSA. A systematic search collected and reviewed all available and relevant published and grey literature (2000–2017). Following removal of duplicates and application of exclusion measures, 46 records remained, which represented 18 of the 49 SSA countries. These records were subsequently charted and thematically analysed.

**Results:**

Three themes were generated; ‘*The Prison Regime’; ‘Navigating inside the Prison Health Infrastructure’* and *‘Accessing the outside Community and Primary Care Health Services’.* Women in SSA prisons experience the same substandard nutrition, overcrowding and unhygienic conditions which exacerbate poor health and infectious disease transmission as males. Human rights abuses, substandard prison conditions and poor access to prison based and community clinical care, along with the invisible nature of women and that of their unique health needs are deplorable.

**Conclusions:**

The review has highlighted the dearth of gender specific strategic information on women prisoners in the region, appalling environmental conditions and prison health care provision, and violation of human rights for those incarcerated. Enhanced donor support, resource allocation, prison health and population health policy reform, health systems surveillance and gender sensitive prison health service provision is warranted. This will help address women prisoners’ conditions and their specific health needs in SSA prisons, and ultimately bridge the gap between prison and population health in the region.

**Electronic supplementary material:**

The online version of this article (10.1186/s12914-018-0170-6) contains supplementary material, which is available to authorized users.

## Background

Globally women and girls constitute a small minority of the total prison population. More than 500,000 women and girls are held in prisons and other closed settings, both as sentenced prisoners or as pre-trial detainees [1]. This number has increased by about 50% since the year 2000 and is rising in all continents. Until recent times according to World Health Organization (WHO), the small proportion of women prisoners have been required to cope with similar provisions and routines as male prisoners. This situation has culminated in a disregard for the distinct and complex needs of women prisoners and neglect of their human rights. Women prisoners constitute a minority of the prison population, and their special health needs relating to gender sensitivity, reproductive health, their children and particularly the treatment of infectious diseases are often neglected [2], unmet by prison health services and compromised by the dominant *‘male’* prison environment [3, 4]. Generally, women have more specific health needs and conditions than male prisoners [5, 6]. They subsequently incur a greater draw on health provisions in prison based health services compared to males [1]. Research where available and generally from high income countries shows that women prisoners also experience greater levels of physical and sexual abuse [5], and greater levels of physical and mental disease [7–10] than non-incarcerated women.

Like all persons, prisoners are entitled to enjoy the highest attainable standard of health. Adequate health services for women in prisons remain mandated under the Sustainable Development Goal’s (SDG) *3, 5,* and *16*, the United Nations Standard Minimum Rules for the Treatment of Prisoners (Nelson Mandela Rules) (A/RES/70/175, *Rules 2, 24, 26 and 32*), the Bangkok Rules for Female Prisoners (A/RES/65/229), the United Nations Convention against All Forms of Discrimination Against Women (*Article 12),* the Basic Principles for the Treatment of Prisoners (*Principle 9*) the Body of Principles for the Protection of All Persons under Any Form of Detention or Imprisonment (*Principle 5),* the International Covenant on Economic, Social and Cultural Rights (*Article 10)* and the Bangkok Rules for Female Prisoners (A/RES/65/229). *Rule 2* of the Nelson Mandela Rules states that in applying the principle of non-discrimination, prison authorities shall consider the individual needs of prisoners, particularly the most vulnerable. *Rule 24* of the Nelson Mandela Rules mandates that provision of health care for prisoners is a state responsibility, ensuring that prisoners should enjoy the same standards of health care as those available in the community. The Bangkok Rules [11] specifically stipulate required standards for management of incarcerated women’s specific health needs and state that women prisoners must be comprehensively screened for health problems and their unique health needs identified on arrival in prison. However, complexities lie in the responsibilities for provision of adequate health care to women prisoners, in terms of equitable quality and access. These complexities can contribute to operation of prison health care services in isolation from public health services, and compromised service delivery, quality and access for women prisoners [1].

### Sub Saharan Africa

Prisons in Sub Saharan Africa (SSA) have seen an increase of 22% in women prisoners in recent years [12, 13]. Women constitute between 1 and 4% of the total SSA prison population [14]. Appalling physical conditions are caused by overcrowding due to high rates of pre-trial detention, poor infrastructure and weak health and criminal-justice systems. Environments are characterized by staff and inmate physical and sexual abuse, food insecurity, and lack of sanitations, with compromised access to health care services exacerbating spread of infectious disease such as human immunodeficiency virus (HIV) infection and tuberculosis (TB) in SSA prisons [7, 15–18]. HIV prevalence in SSA prisons is estimated to be between two and fifty times that of non-prison populations [7] with TB prevalence, six to thirty times that of national rates [19, 20]. In relation to the HIV epidemic, of most concern is that female sex is associated with prevalent HIV infection in SSA prisons [17].

Provisions for women in SSA prisons anecdotally fall far short of the equivalence care standards which are mandated by the aforementioned human rights and international recommendations, and in the recent agreements set out in the Southern African Development Community (SADC) Minimum Standards for HIV in Prisons. This is despite the African Charter on Human and Peoples’ Rights on the Rights of Women in Africa (2003) passed by the Organisation of African Unity, which stipulated that women held in detention were to be held in an appropriate environment for their condition and treated with dignity. Equally, the SADC Protocol on Gender and Development committed regional Member States to ensure provision of adequate nutrition, hygiene and sanitary facilities for women in prisons.

A comprehensive overview of what is known about women prisoners’ health in the SSA region has been called for in the past decade [21, 22]. Research activity on prison health is historically of low priority in the region. The gathering of strategic prison health information is generally restricted to infectious diseases such as HIV/TB, and primarily focused on incarcerated males, and with little direct consultation with women prisoners [6, 18, 22–25]. We aimed to address the need for a comprehensive overview on women prisoners’ health in the SSA region, by undertaking a comprehensive and systematic mapping exercise to establish what is currently known about women prisoner’s health, their health care experiences and their unique prison health care needs when incarcerated in SSA.Stan.

## Methods

Scoping review methodologies have become an increasingly used and valid approach across a variety of disciplines in recent years [26–30]. This type of review is defined as a form of research synthesis that aims to ‘*map the literature on a particular topic or research area and provide an opportunity to identify key concepts; gaps in the research; and types and sources of evidence to inform practice, policymaking, and research’* [28]. The rationale for adopting this method, is that scoping reviews are useful when a topic has not been extensively reviewed [31] (as is the case regarding women prisoners and health needs in SSA) and in order to provide a comprehensive descriptive overview of available information across a wide range of study designs and methodologies [27, 29].

The scoping review method was deemed rigorous and transparent in terms of its step by step protocol to identify and analyse all relevant available sources of information [27, 32] pertaining to women prisoner’s health and their unique health care needs in SSA prisons. The iterative six stage process developed by Arksey and O’Malley (2005) was adhered to throughout and consisted of the following key stages; (1) identifying the research question, (2) identifying relevant studies, (3) study selection, (4) charting the data, (5) collating, summarizing and reporting the results, and (6) an international expert advisory review exercise.

The review process commenced with the establishment of the joint author team, who have public health, gender and human rights, prison health, scoping and systematic review expertise, and have extensive experience undertaking public health research and evaluations in the SSA region [29]. The underpinning research question was; *‘What is known about women prisoners’ health experiences, unique prison health care needs and health care outcomes in Sub Saharan Africa?’* The term “*prison*” was defined and adopted as representing facilities housing both on-remand female prisoners (including jails, police holding cells, and other detention centres) and convicted female prisoners in SSA. Detailed search terms were subsequently generated by the team. See Table 1.

**Table 1:** ‘Search terms and strategy’

Keyword	Alternative	
Prisons	Female inmate*, OR female prisoners *, OR women inmates *, OR women prisoners *, OR health services *,	
Research evidence	health services availability *OR health services accessibility *, OR physical environment * OR physical structure*,	
African Countries	Sub Saharan Africa*OR Africa*OR and the names of all the individual countries classified as Sub Saharan Africa	
1	Prisons	
2	female inmates OR female prisoners OR women inmates OR women prisoners	
3	health services) OR health services availability) health services accessibility OR physical environment) OR physical structure) AND	
4	Sub Saharan Africa OR Africa	
Databases were searched using the appropriate subject headings and/or keywords or text words for the above search groups:		
Sample Search (Pubmed) searched on 1-12-2017		
#	Searches	Results
1	Prison inmates	17477
2	female inmates female prisoners	1185
3	Female inmates OR female prisoners OR women inmates OR women prisoners health services) OR health services availability) health services accessibility OR physical environment) OR physical structure) AND Sub Saharan Africa OR Africa 52	

Key search terms used were *“prisons”, “female inmates,” “female prisoners,” “women inmates,” “women prisoners” female “health services” “health services availability,” “health services accessibility,” “physical environment,”* and *“physical structure”.* These terms were combined with ‘*Sub-Saharan African region’*, and the specific 49 SSA countries .Table 2.

**Table 2:** ‘Sub Saharan African (SSA) countries’

Angola	Côte d’Ivoire	Madagascar	Seychelles
Benin	Djibouti	Malawi	Sierra Leone
Botswana	Equatorial Guinea	Mali	Somalia
Burkina Faso	Eritrea	Mauritania	South Africa
Burundi	Ethiopia	Mauritius	Sudan
Cameroon	Gabon	Mozambique	Swaziland
Cape Verde	The Gambia	Namibia	Tanzania
Central African Republic	Ghana	Niger	Togo
Chad	Guinea	Nigeria	Uganda
Comoros	Guinea-Bissau	Réunion	Western Sahara
Congo (Brazzaville)	Kenya	Rwanda	Zambia
Congo (Democratic Republic)	Lesotho	Sao Tome and Principe	Zimbabwe
	Liberia	Senegal	

The search was restricted to the timeframe 2000 to 2017, and conducted in December 2017 using the University of Zimbabwe and Liverpool John Moore’s University Library catalogues. Following an initial exploratory search conducted by the team, comprehensive searches were conducted in the Cochrane Library, PubMed, EBSCO, Host, Science Direct, EMBASE, Medline, Embase, Medline in Process, PsycINFO and CINAHL. The search included grey literature such as international and national policy documents, thesis and online reports, PubMed Clinical Queries, and Scopus. No limitations on language were applied. Follow up search strategies included hand searching of international aid and development organisations, health, medical and human rights related databases and websites of various government and non-governmental bodies, relevant conferences, and prison and health news sites in each SSA country. Reference lists were also manually searched by the team to identify any relevant studies or literature not captured.

**Fig. 1:**
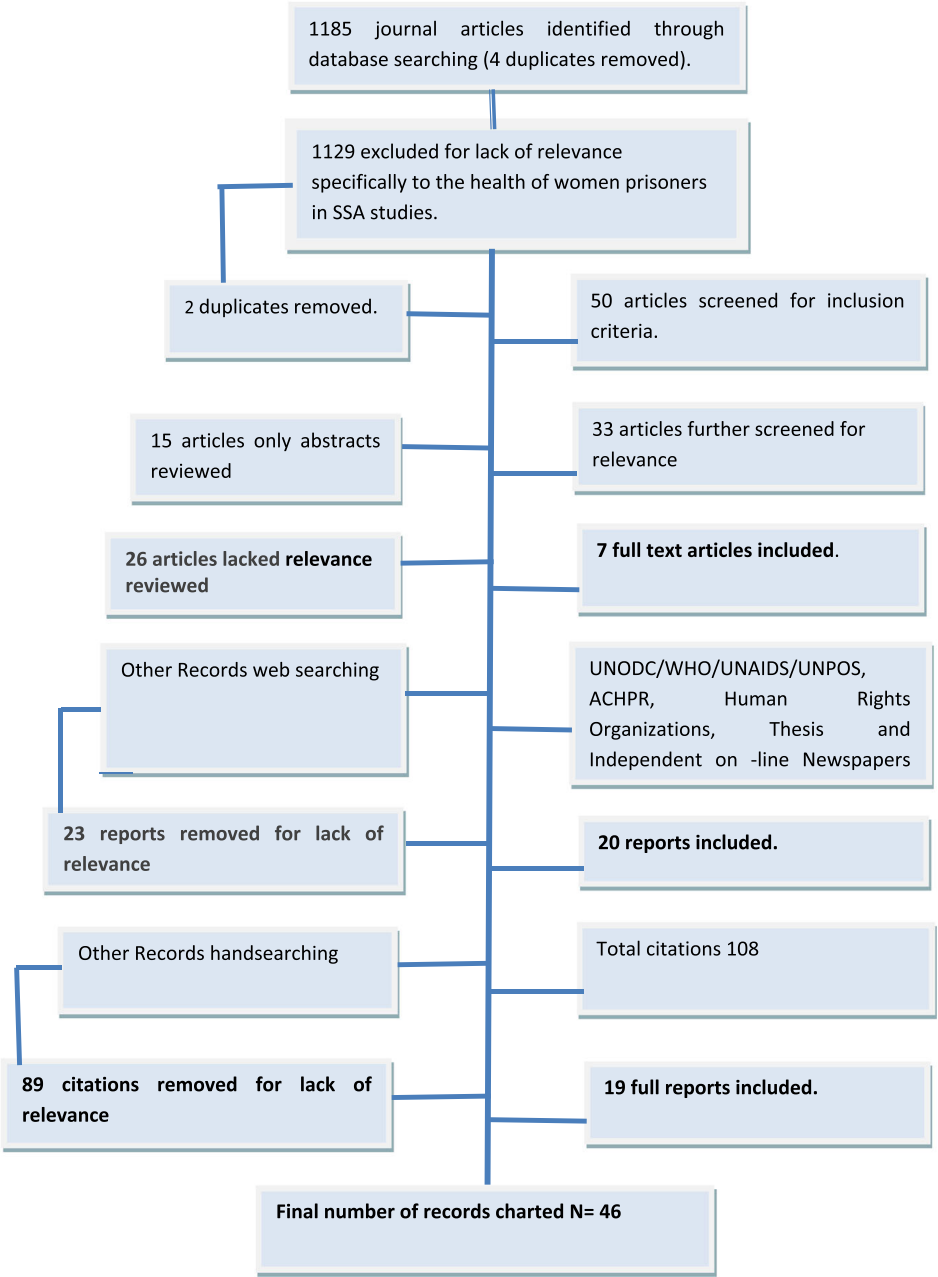
‘Flowchart for inclusion and exclusion of literature’

Citations were managed using the bibliographic software manager EndNote, with duplicates removed manually. The title and abstract of each citation were screened by the second author, and where any doubt remained in terms of inclusion both authors reviewed the citation [29]. Eligibility criteria for inclusion in the review centred on whether citations mentioned women prisoners’ health experiences, unique prison health care needs and health care outcomes in SSA or contained health related content directly relating to women in prisons in the region. To enable the broadest picture of current knowledge and perceptions relating to the issue of women prisoners’ health in SSA prisons, conference proceedings, thesis, commentary pieces and editorials, in addition to empirical data were included. Where possible, any relevant reports on women prisoners in the SSA region providing information about prison staff experiences and perspectives were included. All citations deemed relevant following this screening, were procured for review of the full text version. A second screen of the full-text of each record by the team ensured that records were relevant to the focus of the scoping review. Records were excluded at this stage if found not to meet the eligibility criteria. Figure 1.

Following application of exclusion measures, 46 records were charted and thematically analysed. The charting exercise was conducted by the team (as per Levac and colleagues [29]) which generated specific themes pertaining to women prisoner’s health, their health care experiences and their unique prison health care needs in the SSA region. Charting involved collecting and sorting key pieces of information from each record. A spreadsheet was created to chart all relevant data (data collection categories included year of publication, author, location, method and aim, key findings and conclusion), to enable the identification of commonalities, themes, and gaps in the literature. The team conducted a trial charting exercise as Daudt et al. [28] recommended which involved charting of several records, followed by a team consultation to ensure consistency with the research question and the purpose of this scoping review. Based on this preliminary exercise, the team developed prior categories which guided the subsequent extraction and charting of the data from the records. All records were charted and analysed by both team members in consultation. Disagreements around theme allocation were resolved through team discussion on two occasions. Where additional data extraction categories emerged, team consultation guided decisions around reporting. The charting process concluded with an expert advisory consultation with key experts (medicine, community development, public health and international aid) from the SSA region [27, 28], to ensure no useful records were missed and in order to support and contribute to extraction of multifaceted perspectives from the extant literature.

## Results

The scoping review revealed a limited evidence base pertaining to women prisoners’ health, their health care experiences and their unique prison health care needs when incarcerated in SSA. Literature was found in only 18 of the 49 SSA countries. See Table 2. These countries were South Africa, Uganda, Mozambique, Chad, Zambia, Nigeria, Ghana, Cameroon, Central African Republic, Benin, Malawi, Ethiopia, Namibia, Kenya, Botswana, Somalia, Mali and Zimbabwe. Three main themes were generated ‘*The Prison Regime’; ‘Navigating inside the Prison Health Infrastructure’* and *‘Accessing outside Community and Primary Care Health Services.* We present illustrative quotes from qualitative studies undertaken with women prisoners and prison staff in female prisons where possible. Additional file 1: Table S2.

### Theme one: The prison regime

#### Overcrowding and lack of sanitation

Studies conducted in Zimbabwe, Malawi, Nigeria, Central African Republic, Benin, South Africa, Zambia, Ghana, and Cameroon described physical conditions for women in prisons as inhumane, filthy, overcrowded, poorly ventilated, and with inadequate hygiene and sanitation [6, 16, 21, 33–45]. The African Union Special Rapporteur on Prisons, Conditions of Detention in Africa also observed similar poor conditions in seven SSA countries including Namibia, Uganda, Mozambique, Malawi; Cameroon, Ethiopia and South Africa [46–52]. A Human Rights report [41] in 2010 on Zambia, indicated poor living prison conditions*: “…Our cells are normally stuffed. There is no ventilation, no windows. The sick and healthy are mixed up.”* A Zambian female prisoner described overcrowding and poor ventilation, and the heightened risk of infection spread; *“…You know the cells are currently very full. So if I have TB, coughing all night, and am in the same room with those that don’t have it, they will eventually catch it …”* [22].

Poor toilet facilities and lack of hygiene for women was reported in prisons located in Namibia, Uganda, Mozambique, Malawi; Cameroon, Ethiopia; Botswana, Zimbabwe, Nigeria, Kenya, Chad, South Africa and Zambia [38, 40, 45–51, 53–56]. A South African former prisoner (female) observed poor sanitation and inadequate availability of toiletries; *“…No tissues, no cleaning stuffs, they came after a long time. And you can get germs from the toilets. You can get sick. How are you going to clean the toilets? Imagine 50 or 60 people in one toilet…”* [40]. A Zimbabwean female prisoner described the lack of toilets, with prisoners using 25 l plastic containers *“…There was no toilet facility in the cell… we used a 25-litre plastic bucket. By morning the bucket will be a total mess…”* [33]. In Cameroon overflowing buckets in cell corners were used as the only form of toilet for women, while in Nigeria female prisoners complained of having to use their hands and buckets to remove faeces from overflowing suck-away, and described being exposed to faeces in the prison yard [44, 45]. In Mozambique women used bags to relieve themselves at night [57].

Supply of cleaning materials and soap was described as inadequate in prisons located in Namibia, Uganda, Malawi, Cameroon, Ethiopia, Zimbabwe, South Africa, Chad, South Central Somalia/Mogadishu; Nigeria and Zambia, [6, 22, 33, 34, 36, 39, 40, 42, 46–51, 53–55, 58]. Charitable activity in these countries would (where possible) provide soap and sanitary wear, but with supplies generally insufficient and inconsistent. The unavailability of soap for women prisoners in Zambia and reliance on family support for basic sanitation was observed; *“If others don’t bring them for us, we have nothing. There are lots of people with no relatives here. They have nothing…”*[42]. Regular water shortages also affected women prisoners’ hygiene in prisons in Namibia, Uganda, Cameroon, Ethiopia, South Africa, Nigeria, South Central Somalia/Mogadishu, Zambia and South Africa and Zimbabwe [6, 22, 36, 38–40, 44–47, 51, 56, 59].

### Dealing with menstruation

A lack of prison based support for women’s needs around menstruation was reported in prisons located in Namibia, Uganda, Mozambique, Malawi; Cameroon, Ethiopia, Zimbabwe, Zambia, Nigeria and South Africa [6, 33, 37, 40, 42, 44–51, 58, 60]. Zambian women prisoners described getting ad hoc supplies of disposable sanitary pads through family, friends or churches [22]. Zimbabwean women prisoners described using newspapers, tissues, pieces of blankets, and sometimes prison uniforms in the place of sanitary pads [33]. A former prisoner shared her experience on inadequate sanitary wear during menstruation; *“…We ended up tearing blankets on the fence… the material was very rough, it grazed our skin, some used newspapers by rubbing them to make them soft and absorbent… others bathed frequently during the day…”* [33]. Ethiopian and Malawian female prisoners washed and reused a prison provided cloth for menstruation, but were not provided with soap [37, 51]. In South Africa, prison officials were reported to demand to see evidence of soiled sanitary towels, before issuing replacements [40]. Unhygienic disposal of used sanitary pads was also reported in Malawian, Zimbabwean, Zambian and South African female prisons [22, 33, 37, 40].

#### Poor nutrition

Poor quality nutrition and inadequate daily provision for women prisoners (and their children) was described in Uganda, Mozambique, Malawi, Nigeria, Mali, South Africa, Benin, Zimbabwe, Zambia and Cameroon. This was especially the case for those who were pregnant, those nursing and those with young children. Women’s dietary needs were not considered in prisons located in Malawi, Nigeria, Kenya, Chad, Cameroon, Zimbabwe and Ghana [35–38, 40, 43, 44, 54, 55]. In Zambia, nursing mothers living with HIV were provided with nutritional supplementary feeding but not consistently [56]. In contrast, South African women prisoners with health conditions (pregnancy, lactating, daibetes, Acquired Immune Deficiency Syndrome (AIDS) and those of certain religions could make requests for special and better-quality diets and these were generally granted and the same observation was made in Ethiopia for expectant mothers, nursing mothers, children and sick prisoners (Agboola 2016; ACHPR 2004a) [40]. Two women in Zambian prisons shared their experiences and said; *“…I think with the kind of food that we eat, I can develop illnesses. The fish we eat has a lot of stones and the mealie-meal we use is not well taken care of …”* and *“…There is no food here. There is no proper food. We only eat the stony kapenta every day. Even rice, there are many of us, yet there is only a small pot …”*[22]*.*

#### Physical and sexual abuse by prison staff and inmates

Within the prison regime, the threat of physical and sexual gender violence is common for women prisoners. Women prisoners in SSA are at extreme risk of physical abuse by other female inmates, and by police and prison officials [61–63]. Physical and sexual abuse perpetrated by police and prison officers were reported in Malawi, Zambia, and Nigeria [6, 42, 49, 58, 60, 63]. Vulnerability to sexual abuse and sexual exploitation was reported in South Africa, Zambia and Nigeria [58, 60, 64, 65]. A Zambian female prisoner described the risk for transactioning of sex in exchange for release; *“…They arrested and they beat me, asking questions. They didn’t sexually abuse me, but they asked me to have sex with them. They said they would release me if I did, and I said no…”* [42]. Nigerian women were reportedly engaging in risky sexual behaviours when in prison and described as vulnerable to sexually transmitted infections (STIs), HIV and exploitation [58].

### Theme two: Navigating inside the prison health infrastructure

#### Inadequate prison based health service provision in female prisons

Current provision of prison based health care for incarcerated women in SSA is inadequate, fails to meet their distinct sexual and reproductive needs, and falls far short of what is required and mandated by human rights and international recommendations [1, 21, 44]. Fifteen SSA countries (Namibia, Uganda, Mozambique, Malawi, Cameroon, Botswana, Nigeria, Mali, Kenya, Benin, Zambia, Chad; South Central Somalia/Mogadishu, Ghana and Zimbabwe) reported on inadequate prison based health services for women [16, 21, 35–38, 42–50, 53–57, 59, 66]. Sub-standard and ill equipped health and clinical services provision was characterised by essential medicines stock outs, lack of trained health personnel (or limited to restricted opening hours for women), lack of routine medical check-ups for women, limited availability of equipment or lack of basic investigation equipment (for example functioning sphygmomanometers, thermometers, poor coverage of gynaecological care (cervical smears, breast examinations), absent or gender insensitive prison health care policies and standard operating procedures (SOPS) for women. In Zambia, Mozambique, Ghana, Nigeria and South Africa, women prisoners were reportedly treated with any available medication and not according to SOPS [16, 40, 43, 57, 58]. In Ghana, the prison health care provider attributed the recurrence of candidiasis among female prisoners due to erratic availability of metronidazole, and reported lack of medicines for the treatment of simple ailments like malaria [43]. In Ghana and Zambia, female prisoners were reported to be disadvantaged clinically, in comparison to males who were medically prioritised and treated [43, 56]. Women prisoners in Zambia and South Africa self-medicated with diverted medicines [22, 40]. One former prisoner in South Africa remarked; *“…I never got any medicine from the Kas [clinic] while I was there [in prison]. I had an abscess in my mouth… I had to buy antibiotics from another woman [fellow inmate] who was taking antibiotics for her tooth that was removed...”* [40]. Improvised self-management in South Africa was also common. *“…For colds, we usually make a mixture of… hot water, a spoon of maple syrup, a spoon of lemon juice, and two tablets of crushed… You learn survival skills in there …”* [40]. Lack of adequate medicines was reported by women in Mozambique prisons, with the doctor reportedly treating every aliment with paracetamol, and not adhering to clinical SOPS for the different diseases [57]. With exception of South Africa, that provided minimal mental health services for women prisoners, other SSA countries did not provide adequate female mental health supports, despite high rates and occurrence of post -traumatic stress disorder, depression and self-harming among incarcerated women [1, 21, 40, 61].

#### Access to prison based health clinics

Eleven SSA countries reported limited prison based health services for women, and poor access for women to prison based clinics in Namibia, Uganda, Malawi, Cameroon, Ethiopia, Nigeria, Zambia, Chad, Mozambique, Ghana and South Africa [16, 37, 38, 40, 42, 43, 45–47, 49–51, 55, 56, 58]. Under resourcing of prisons by governments affected provision and access to health care by female prisoners in Cameroon and Zambia [22, 44, 56]. Pooling of resources across Zambian and South African prisons resulted in the systematic de-prioritisation of women’s health care [22, 41]. In Zimbabwe, a senior prison administrator described the lack of basic resources such as decent accommodation for women prisoners, inadequate food and availability of gender specific health services, and attributed this to insufficient funding from the fiscus [35].

In four SSA countries (Kenya, Zambia, Mozambique and Cameroon) restricted prison-based health facility operational hours for women (with priority and majority of days allocated for males) impacted on their ability to access care when needed, and their uptake to clinical care [22, 42, 45, 54]. A pharmacy operated as a health clinic/centre in a Mozambiquen prison, but with restricted operational hours for women, Monday to Friday from 9 am-3 pm [57]. A female detainee shared her experience, “… *If one gets sick after three o’ clock in the afternoon one had to wait until the next morning and nothing is going to change because the only thing he is going to give is Paracetamol. A prisoner needs to pray to God to not get sick from Friday until Monday…”* [57]*.*

Equity in prison health care access in Zambia was reported to be affected by prisoners’ hierarchy (older inmates had an advantage of accessing health services when in need compared to newly incarcerated prisoners) [22]. Female prisoners in Zambia reported that long working hours frequently prevented them from accessing necessary medical care; *“…It is not possible here to go to the doctor. At the moment we wake up, we go to the field, then we go to a different field. Even if you complain [that you are sick], the officers tell you that you still have to go…”* [16]. Lack of privacy and confidentiality during consultations included negative staff attitudes that were described as additional barriers to women’s attempts to access prison based health services in Namibia, Uganda and Zambia [22, 46, 47].

#### Prison based sexual and reproductive health Services for Women

Provision of specific sexual and reproductive health services for women prisoners in the SSA region were reported as poor, ill-equipped and not meeting recommended international standards [1, 37, 42, 61]. Ante-Natal Care (ANC) and Post-Natal Care (PNC) were reported to be non-existent in prisons located in Malawi, Zambia, Cameroon, Ghana, Nigeria and Zimbabwe [22, 35, 37, 42–45, 56, 58]. ANC and PNC was not routinely provided in all Zambian prisons, and where it existed, did not meet international standards [42] and with provision of ANC/PNC for women in Ghanan and South African prisons depending on the type of sentence [40, 43]. Women in Ghanan prisons were generally able to access ANC, delivery and PNC services outside of prison in community services [43]. A different study observed that in Zambia, limited access to basic reproductive and preventive health services was provided; and where it was possible pregnant mothers gave birth outside of the facility, either at a public health centre or hospital [56]. One pregnant Zambian prisoner said; *“…I have not been to the clinic yet, no antenatal care. I went to the clinic once but was told the nurses were not working...”* [42]. Accounts of women delivering in cells were reported in Zimbabwe [21]. An ex-inmate in Zimbabwe [35] shared her experience on lack of availability and access to ANC, delivery facilities and PNC in prison and the negative attitudes of health providers at referral institutions in Zimbabwe; “…*We had no regular medical check-ups… And then there was the abuse- They called us names…Some of the nurses shouted at me while in labour at referral hospitals where I had been taken in my prison uniform and handcuffs… You are forced to return to jail within 48 hours after giving birth at public health facilities together with the newly born baby and that is when you get an extra blanket for the baby...”*[35].

#### HIV prevention, treatment, care and support for women prisoners

HIV Prevention, Treatment, Care and Support (PTC&S) and treatment for opportunistic infections such as TB in SSA is not adequately implemented within prison based health services [15, 62]. HIV testing, TB screening and treatment coverage in SSA prisons is weak, with limited or no provision of HIV prophylaxis for prevention of mother-to-child transmission (PMTCT), and further compounded by equally limited access to highly active antiretroviral treatment (ART). Zambian women prisoners were less likely to have been tested than males, despite mandatory HIV testing of all pregnant women, *“…For those who are pregnant, they are tested for HIV...Whether you like it or not you are tested to prevent transmission to the baby…”* [42]. Medical care in Malawian, Malian and Zambian prisons was reportedly administered to women prisoners, once the AIDS condition had deteriorated and was generally treated as medical emergency [37, 42, 66]. There is an identified programming gap in service provision and accessibility of HIV, PTC&S for women and their children [18]. Other studies describe access depending on prison staff, with prison warden’s negative attitudes impacting on access to ARVs [17]. A female detainee who experienced challenges in accessing ARVs while in detention in Rwanda shared her experience, *“…I normally get my medicine once a month and I take it each day. I started ARVs in 2006, but when I was in Kwa Kabuga I did not get them…”* [17]. Non-availability of ARVS was reported in Uganda by another female detainee who remarked, “…*When I told the prison officer I was HIV positive, he said, ‘Fight on, complete the sentence, go home, and get treatment.’ It meant he can’t do anything for me. There were wardens I informed. They said prison has nothing to offer me...”* [17]. In South Africa initiation and adherence to ARVs was impacted by legislation that prohibited nurses from prescribing medicines without authorisation, and which was described as contributing to delays in commencing therapy and chronic-disease management [17].

Additionally, prevention efforts were observed to be weak with nurses lacking in training of preventative medicine and insufficient clinical staff in prisons in Malawi, Tanzania, Swaziland, South Africa, Mauritius and Zimbabwe [65]. Other studies described lack of national resource allocation to prisons as a barrier to HIV/TB management [65]. In Mali Voluntary Counselling and Testing (VCT) was not routinely provided and female prisoners cited lack of confidentiality by health providers, stigma and awareness of low ARV availability as barriers to uptake of VCT [66]. A review with 5 case studies in SSA underscored the challenge of assessing the magnitude of the burden of disease of TB among women prisoners, which is reportedly compounded by lack of data disaggregation [17].

### Theme three: Accessing the outside community and primary care health services.

#### Inadequate prison based resources to support outside care

Bureaucracy in administrative procedures, requirements to procure resources for transport and fuel for medically referred prisoners, lack of adequate trained health personnel and prison staff for the transfer of sick inmates, inadequate vehicles for transportation and fuel, and security fears were described by prisoners and prison officers as delaying clinical care (in some instances for several weeks after falling ill) outside of prisons located in Namibia, Malawi, Cameroon, Mali, Chad, Mozambique, Ghana and Zambia [22, 43, 46, 49, 50, 55–57, 66]. As one prison officer in Zambia commented; *“…There are no resources, when you have to take prisoners to the hospital, …then you are wondering how you are going to help these people? So that makes me dread work … even fuel you have to go and beg from other services …”* [22]. Several instances were described in Namibia, Malawi, Uganda, Cameroon and Zambia where women prisoners were told by prison officers that they would not be taken to the hospital unless they themselves were able to raise the fuel cost [16, 42, 46, 47, 49, 50].

#### Accessing emergency and chronic care outside of the prison

In some prisons in Zambia, access to outside health care was reported to be controlled by medically unqualified and untrained prison officers [42]. Negative staff attitudes in Zambian prisons was also observed to have serious implications for women prisoners emergency access to care, as well as for continuity of care for those with chronic conditions such as TB and HIV/AIDS [22]. This was particularly problematic at night. In Zambia, a prisoner remarked *“…There are delays in getting to the clinic. It depends on the officials, if they want to take you there or not. Sometimes you can go as long as a month waiting to go to the clinic.... They don’t open the door of the cell at night for anything…” .*[42]*.* Issues relating to lack of confidentiality and privacy for women when accessing outside clinical care were described, particularly in relation to prison security protocols that required prison officers to sit in with patients during primary care and hospital consultations in Mali, Ghana and Zambia [22, 43, 66]. Additionally, in Mali [66] reported that there was no proper follow up of prisoners along the continuum of clinical care due to lack of transport to attend routine appointments.

## Discussion

The scoping review represents a unique and first step toward gathering mapping available literature on women prisoners’ health experiences, unique prison health care needs and health care outcomes in SSA. Scoping reviews offer a novel route to synthesising a wide range of literature specific to women’s health and health care provision in SSA prisons. We have provided an extant descriptive overview of the situation for women incarcerated in SSA prisons. The review was thorough in terms of its multi layered strategies to locate information. This occurred via online searches, hand searching of international aid and development organisations, health, medical and human rights related databases, websites of various government and non-governmental bodies in SSA, relevant conference listings, and prison and health news sites in each SSA country. In addition, manual searching of reference lists, and the expert consultation post charting occurred in order to cross check for any missing data sources. Limitations of the review centre on the relative lack of available data sources and published studies on women prisoners, with only 18 countries out of the 49 Sub Saharan African countries represented in the final data set. The gathering of strategic information and investment in academic research in SSA prisons at country level warrants improvement.

The healthcare needs and experiences of female prisoners in the SSA region remain de-prioritised and inequitable in prison health policies, prison-based health services, and SOPS, and yet are also poorly understood and rarely studied. This, and the dearth of gender disaggregated and empirical information available on women prisoners’ health, and particularly regarding sexual and reproductive health, and infectious disease prevalence [6, 22, 43, 67, 68] is deeply concerning and occurs within the context of human rights violations of incarcerated women in SSA prison environments. Despite global commitments to end HIV and TB in the SSA region, most SSA countries in 2017 still do not collect or report comprehensive information about the incidence, prevalence, or clinical outcomes of HIV infection, TB and AIDS in incarcerated populations [17].

The review underscores how SSA prison environments remain contra not only to international mandates, but also regional agreements in the SADC Minimum Standards for HIV in Prisons; the Protocol to the African Charter on Human and Peoples’ Rights on the Rights of Women in Africa; and the SADC Protocol on Gender and Development. Human rights violations, abuses and substandard prison conditions, along with the invisible nature of women and that of their health are deeply concerning [6, 69]. In terms of health care, the current situation in SSA prisons for incarcerated women is contra to all available guidelines for women in prisons [70], which mandates provision of safe and appropriate health services (including sexual and reproductive, gynaecological and dental clinics); the presence of skilled health personnel trained to follow universal precautions guidelines in prevention of HIV transmission through medical practices (injections, procedures or examinations); and the provision of equivalent health services to those available in the community. SSA countries are encouraged to enhance provision of internal prison based health services and trained health personnel in prisons specialized in women’s health, and at the very minimum provide equivalence of care to that provided outside of incarceration, in line with international mandate United Nations Rules for the Treatment of Women Prisoners and Non-Custodial Means for Women Offenders (the Bangkok Rules) [11]. Of note are the various very useful UNODC, UNAIDS, WHO and ICRC technical guidance and checklist documents available such as *‘Fast Track to end AIDS by 2030: For People in Prisons’*, UNODC, ‘*Women and HIV in Prison Settings’,* UNODC/UNAIDS, ‘*Women’s Health in Prisons’*, WHO/UNODC, and *‘Health Systems and Needs Assessment in Prisons - Practical Guide and Toolkit’,* ICRC*.*

Despite the proportion of women prisoners in SSA prisons being lower than that of their male counterparts’, they are distinctly and negatively affected by prison environmental factors, which impact severely on their specific female health needs and wellbeing [21, 22, 38, 53]. The appalling SSA prison environments and regimes all serve to exacerbate poor women’s physical and mental health and the transmission of communicable diseases (such as HIV and TB). Women prisoners in SSA experience unique gender specific health challenges relating to menstruation and lack of appropriate sanitary towels, pregnancy and childbirth [6, 18, 21, 42, 71]. WHO [4] currently recommends that disposal possibilities for used sanitary towels are freely available and easily accessible to women in prison at all times. This is not the case in the SSA prisons. Internal prison health infrastructures in the SSA region are characterized by weak responsiveness by female officers to prisoner requests for healthcare, untrained health personnel and lack of medicines. Accessing of outside community health for women prisoners (and their children) are hindered by prison procurement policies, low resources, inmate hierarchies, lack of suitable medical transport, fuel and prison staff to accompany medical referrals, and general lack of access to sexual and reproductive health and child health services.

The issue of HIV/AIDS in SSA prisons is both a human rights and public health issue, which requires a strategic approach with shared public health and human rights goals in policies, to prevent HIV transmission and improve health for all, whilst at the same time ensuring equivalence of care to that provided outside prison, with the respect of human rights and dignity of those infected and requiring treatment [72]. It underscores the imperative that women prisoners are advocated for as a priority risk population and SSA prisons must continue to contribute positively to broader efforts to control communicable diseases in the general population. This is vitally important given prison release and the return of women and their children to their communities, and one that is cognisant of their distinct vulnerabilities as HIV/TB risk population and the continuing disproportionate level of HIV/AIDS affecting women and girls in the SSA region [15, 18, 68, 69].

It is paramount that SSA governments, national prison and health policy makers, international organisations, donor and NGOs collaborate and advocate for prison health policy reform and women’s health guideline development to ensure women prisoners and their health needs are not neglected. This is consistent with the UN Sustainable Development (SD) agenda of ‘*no-one left behind’*. Health citizenship of women prisoners must be fostered along with a sense of belonging to a community of people whose citizenship has been devalued at two levels, namely that of gender, and that of incarceration. Such prison health policy reform activities sit within the global aim of leveraging the end of AIDS (Fast Track Approach to end AIDS, [73] (UNAIDS 2016–2021) through working and collaborating in an interdisciplinary and multi-sectoral partnerships (SDG 17) [74] (General Assembly Resolution, 2015) to challenge and address health inequalities of women prisoners.

At the time of writing this review, the Twenty-sixth session of the Commission on Crime Prevention and Criminal Justice (CCPCJ), May 2017, adopted the resolution E/CN.15/2017/L.5/Rev.1, *“Ensuring access to measures for the prevention of mother-to-child transmission of HIV in prisons*”. This resolution requests Member States in close cooperation with UNODC and other relevant United Nations entities and other relevant stakeholders, to increase their capacity to eliminate mother-to-child transmission of HIV within sexual and reproductive health, and support HIV prevention and treatment programming in prisons. Given the connection between prison health and public health, it is vitally important for prison services in all countries particularly high-burden TB/HIV co-infected countries in the SSA region, to provide uninterrupted services for prevention of mother-to-child transmission of HIV to all women in prisons, and to achieve the goal of ending AIDS as public health threat by 2030, *“leaving no one behind”.*

## Conclusion

Women prisoners are entitled, without discrimination, to health care, both within and accessing community care including preventive measures, of a standard equivalent to that available in the outside community [75]. The review has highlighted the environmental conditions, violation of human rights protection for women (and their children), and substandard health care provision for women incarcerated in SSA prisons. It underscores the lack of information, and the urgent need for donor support, national governmental resource allocation, prison and health services policy reform, overall health systems strengthening and the requirement for gender and context specific guidance to better address women’s health needs in SSA prisons. The African HIV in Prisons Partnership Network which was set up in as an outcome of the 2009 the African Declaration of Commitment for HIV and AIDS PTS&C in Prisons across the region could offer a renewed partnership focus on women prisoners health and health needs, and bring together prison and correctional services, prison health systems, public health systems, national AIDS committees, international and national civil society organizations. Efforts to support this network with a gender mainstreaming dimension are warranted given that its current visibility among SSA Member States is weak. Surveillance and investment in research on women prisoner’s health experiences and needs in SSA, as well as political commitment and advocacy at higher policy level is warranted in order to respect women’s rights to adequate prison conditions and access to health care while incarcerated, and ensuring that interventions motivated by public health considerations are respectful of women’s rights at all times.

## Additional file

Additional file 1: **Table S2.** Summary of Records. The scoping review charting of records (DOCX 74 kb)

## Abbreviations

AIDS: Acquired Immune Deficiency Syndrome
ANC: Ante-Natal Care
ART: Anti- Retroviral Treatment
CAT: Computed Axial Tomography
CCPCJ: Commission on Crime Prevention and Criminal Justice
HIV: Human Immunodeficiency Virus infection
MRI: Magnetic Resonance Imaging
PMTCT: Prevention of Mother-To-Child Transmission
PNC: Post-Natal Care
PTC&S: Prevention, Treatment, Care and Support
SADC: Southern African Development Community
SD: Sustainable Development
SDG: Sustainable Development Goal
SOP: Standard Operating Procedures
SSA: Sub Saharan African (SSA)
STI: Sexually Transmitted Infection
TB: Tuberculosis
VCT: Voluntary Counselling and Testing
WHO: World Health Organization
