# Supplementary material for: Contemporary women prisoners health experiences, unique prison health care needs and health care outcomes in sub Saharan Africa: a scoping review of extant literature
Source: BMC Int Health Hum Rights. 2018 Aug 6;18:31. doi: 10.1186/s12914-018-0170-6 (PMC6080512; doi:10.1186/s12914-018-0170-6)
Supplement: Supplementary file 1 — Table S2. Summary of Records. The scoping review charting of records (DOCX 74 kb) [file 12914_2018_170_MOESM1_ESM.docx]

| **Authors** | **Title, Journal, Year of Publication, Volume, Pages** | **Aim** | **Location** | **Method of Study** | **Results** | **Conclusion** |
| --- | --- | --- | --- | --- | --- | --- |
| **Journals** | | | | | | |
| Topp SM, Moonga CN, Mudenda C, Luo N, Kaingu M, Chileshe C, et al. | Health and healthcare access among Zambia’s female prisoners: a health systems analysis  Int J Equity Health. 2016;15:157. | To identify and examine the interaction between structural, organisational and relational factors influencing Zambian women prisoners health and healthcare access. | Zambia | Qualitative Research  In-depth interviews of 23 female prisoners across four prisons, as well as 21 prison officers and health care workers. | In all prisons female inmates reported poor environmental conditions with particular emphasis on poor sleeping conditions and terrible sanitation.  Overcrowding with 8-10 women sharing a single cell meant for inmates per cell.  Insufficient toilets, broken toilets or lack of access to toilets (at night)  Inability to keep toilets clean, through overuse and lack of cleaning products mentioned for all sites.  Frequent, but ad hoc donations of disposable sanitary pads were made by various Church groups at all sites, friends and family.  Lacked of pads and relied on reusable cloth pads hand-made by themselves from cotton wool and scrap cloth donated by the Church.  Unhygienic disposal of used pads while re-usable pads had to be washed and dried daily despite the scarcity of laundry soap.  Insufficiency of prison rations or poor quality of prison food as a health concern.  Long-serving and senior inmates had privileged access to services due to their closer relationship with officers.  Variable and poor access to health services.  Lack of internal health facilities.  Limited availability of officers to accompany women prisoners to external health services.  Access to referral hospitals further away than primary health centres was problematic due lack of transport and fuel and accompanying officers.  Women told by officers to fuel cars using own resources in order to be transported to hospital.  Several officers acknowledged the need to have more inmates sick, thereby justifying the use of the vehicle and expense of the fuel to hospital.  Pooling of resources across the four prisons that formed the larger complex, resulted in less resources for female health care.  Female inmates’ access to the prison clinic was just two days a week, one of which included Sundays when health providers worked truncated hours or occasionally not at all.  Remainder days of the week were allocated to male  Negative reports on prison officers’ attitudes to women prisoners when sick.  Access to health care difficult at night  Structural and attitudinal barriers had serious implications for emergency access to care (such as at night) as well as for continuity of care for those with chronic conditions such as tuberculosis (TB) and HIV /AIDS.  Lack of confidentiality and privacy, particularly in relation to security protocol that required prison officers to sit in on consultations.  Medicines stock outs, limited equipment and a lack of health personnel (or at least limited opening hours) at the adjacent primary health clinics were also frequent problems. | Health seeking behaviour is affected by both structural factors and power dynamics in prison settings. When designing interventions in prison settings targeting women it is critical to address these key fundamental dynamics to promote positive health seeking behaviour |
| Topp SM, Moonga CN, Luo N, Kaingu M, Chileshe C, Magwende G, et al. | Mapping the Zambian prison health system: An analysis of key structural determinants. Glob Public Health. 2017; 12:858–75. | To identify major structural barriers to strengthening the prison health systems. | Zambia | Case-based analysis drew on key informant interviews 7, memos generated during workshops 4 document review | Prisons severely overcrowded.  Lack of adequate food  Non-availability of tape water within prisons.  Female inmates have limited access to basic reproductive and preventive health services.  In some prisons HIV-positive breastfeeding mothers reported being provided with nutritional supplements, but this is inconsistent.  Where possible, pregnant women give birth outside of the facility at a public health centre or hospital.  Provision of sanitary products ad hoc and largely NGO supported.  ANC and PNC not routinely provided due to lack of access and/or resources.  Under-resourcing, lack of personnel and the absence of sufficiently close health services affect female prisoners more acutely.  Health personnel reported difficulties in securing access to a vehicles or fuel to collect medical supplies.  Availability of diagnostic equipment within prison health centres limited | Health access and provision in Zambian prisons was affected by weak governance inadequate human resources and financing. |
| Dixey R, Nyambe S, Foster S, Woodall J, Baybutt M. | Health promoting prisons–An impossibility for women prisoners in Africa? Agenda. 2015;29:95–102. | Interrogation of concept of health promoting prisons as opposed to health provision with a thrust of prevention | Africa | Literature | More female inmates with HIV prevalence (43%) than population 13%  Not much known about health status and needs, let alone female inmates’ psycho-social needs. Nutrition, sanitation, ventilation, hygiene and quality of water poor and inadequate.  Women are underserved by general healthcare programs including those offering TB/HIV testing.  Physical and sexual abuse conducted by police and prison officers which qualify as torture under international law”  Women with no relatives to provide toiletries and food forced to go without and resulting in trading prison work for the most basic of items e.g. food and soap.  Barter trade for survival due to lack of basic toiletries. | There is dearth of information and research on health of inmates focusing on females as available research tends to be gender blind |
| Reid SE, Topp SM, Turnbull ER, Hatwiinda S, Harris JB, Maggard KR, et al | Tuberculosis and HIV control in sub-Saharan African prisons:“thinking outside the prison cell.” J Infect Dis. 2012;205:S265–S273. | To describe the challenges inherent in current approaches to tuberculosis control in prisons and consider the alternatives. | Africa | Literature review | Female inmates face the same overcrowding and unsanitary conditions as males that contribute to the spread of infectious disease and poor health.  Female inmates experience unique health-related challenges, such as menstruation, pregnancy and childbirth, care of children inside and outside prison, and extreme violence and (often sexual) abuse by prison officers and male prisoners | Unconducive prison environment exacerbates the spread of communicable diseases |
| Todrys KW, Amon JJ. | Health and human rights of women imprisoned in Zambia. BMC Int Health Hum Rights. 2011;11:8. | The main objective was to document and respond to specific human rights issues, monitor human rights conditions, and assess human rights protections in Zambian prison. | Zambia | A mixed-methods study including in-depth interviews with 38 adult female prisoners and 21 prison officers in four Zambian prisons Key informant interviews with 46 officials from government and non-governmental organizations and a legal and policy review. | Women prisoners live in conditions of severe overcrowding. Prisons are over 300 % of capacity, inmates sleeping 4 to a mattress, packed together in unventilated cells with young children and the sick  Both prisoners and prison officials reported insufficient and nutritionally inadequate food. Prisoners rely on their relatives for supplementation or trade work for food.  No provision of basic necessities as soap, toothpaste, or sanitary pads.  Of Zambia's 86 prisons, only 15 had any health clinic or sick bay.  For those at prisons without a clinic–and for those with more serious medical conditions at those with a clinic–access to care is controlled by medically unqualified and untrained prison officers.  Both prisoners and prison officers, indicated a lack of adequate prison staff for the transfer of sick prisoners, inadequate vehicles for transportation and fuel, and security fears prevent inmates from accessing medical care outside of prisons, in some cases for weeks after they fall ill.  Inmates reported delays of up to a month waiting to go to the clinic.  Incarcerated pregnant women described inadequate, and in some cases non-existent care.  For some ANC care existed but did not meet international standards.  No PMTCT program under the prison medical directorate, though PMTCT programs have been scaled up in recent years for the general population.  Inadequate nutrition is a serious problem for pregnant women and women with children in prison.  The Prisons Service allocates no food to children who live with their mothers in prison facilities. In situations where women are unable to breastfeed, the prison does not offer infant formula.  HIV testing and treatment was offered at six prisons nationwide with the assistance of an NGO, and as of March 2011, prison-based TB screening and treatment offered only at three prisons nationwide as part of a pilot programme.  HIV testing not mandatory prenatally and Zambia Prisons Service policy prohibits compulsory HIV testing.  Mandatory testing was conducted prenatally for all pregnant women.  Female inmates previously held in police custody, reported physical and sexual abuse | Access to health care by female prisoners was a challenge due to a number of factors identified in the study. Unique female health needs need to be taken into consideration when designing health interventions within prison settings |
| Todrys KW, Amon JJ, Malembeka G, Clayton M | Imprisoned and imperiled: access to HIV and TB prevention and treatment, and denial of human rights, in Zambian prisons. J Int AIDS Soc. 2011;14:8. | To better understand the relationship between prison conditions, the criminal justice system, and HIV and TB in Zambian prisons | Zambia | Mixed method  facility assessments and in-depth  interviews with 246 prisoners and 30 prison officers at 6 Zambian prisons; a review of Zambian legislation and  policy governing prisons and the criminal justice system; and 46 key informant interviews with government and  non-governmental organization officials and representatives of international agencies and do | Some health facilities had very little medical capacity beyond distributing paracetamol  According to staff and prison officers, in prisons without a medical clinic – and for prisoners with more serious medical conditions requiring advanced care - access to care is frequently controlled by medically unqualified and untrained prison officers who evaluate and determine if medical visits to community health facilities are necessary.  Prisoners and prison officials blamed the lack of sufficient prison staff, transportation and fuel.  Security fears, lengthy delays in the transfer of sick prisoners to medical care outside, in some cases for days or weeks after they fall ill.  Inmates consistently reported that the requirement to work long hours in farms frequently prevented them from accessing necessary medical care.  Variability in Voluntary Counselling and Testing (VCT) uptake with less women likely to undertake testing. | There serious barriers to HIV/ TB prevention and treatment that have negative public health implications. Failure to manage these 2 epidemics can result in the development of drug resistance strains and spread of TB in the general population beyond incarceration. Overcrowding in prisons is exacerbated by extended  pre-trial detention and disparities exist among the among different categories of prisoners and  juveniles, women, pre-trial detainees and immigration detainees were less significantly likely to access health services compared to other categories of prisoners |
| Todrys KW, Amon JJ. | Criminal justice reform as HIV and TB prevention in African prisons. PLoS Med. 2012;9:e1001215. | To better understand structural barriers to HIV and TB prevention in African prisons. | Southern Africa  18 SADC countries | Survey | Nearly all SADC African prison administrators cited inadequate funding as the most significant challenge to their ability to deliver health care.  Prison authorities in Tanzania cited insufficient numbers of qualified medical personnel.  Swazi authorities cited poor infrastructure for health, as well as absence of a medical officer trained in HIV treatment.  Zimbabwean authorities cited inadequate equipment, physical infrastructure, and training of health personnel in HIV/TB management;  South African authorities cited shortages of health care professionals and lack of appropriate facilities for the management of communicable diseases;  Malawian officials cited a shortage of medical equipment and drugs;  Mauritian prison authorities noted insufficient health staff.  Significant gaps in the availability of HIV/TB-related prevention and care in Zambia and Uganda. | Lack of key resources necessary for effective and efficient HIV and TB control and prevention negatively affected management of the two epidemics. within the SADC region. Over the years the SADC region has experienced a serious brain drain and lack of capital has affected infrastructural development of prisons |
|  | The HIV and TB Prison Crisis in Southern Africa \| Human Rights Watch [Internet]. [cited 2018 Feb 8]. Available from: https://www.hrw.org/news/2010/07/23/hiv-and-tb-prison-crisis-southern-africa | To highlight the double epidemic of HIV/AIDs and TB | South Africa | Editorial  General description of poor prison conditions in SA and Zambia and how they contribute to the double epidemic | Overcrowding  Lack of food and soap  Prisons are poorly resourced  Lack of financial resources | SSA has a double epidemic in terms of HIV/AIDS and TB prevalence hence the urgent need or measures to curb the spread of both epidemics in SSA prisons. The need to aggressively address the 2 epidemics is in line with the observation prison health is good public health practice |
| Sarpong AA, Otupiri E, Yeboah-Awudzi K, Osei-Yeboah J, Berchie GO, Ephraim RKD | An Assessment of Female Prisoners’ Perception of the Accessibility of Quality Healthcare: A Survey in the Kumasi Central Prisons, Ghana. Ann Med Health Sci Res. 2015;5:179–184. | To explore the background characteristics of female prisoners and how it influences their assessment of the quality of accessible healthcare in the Kumasi Female Prison. | Ghana | Descriptive, cross-sectional study with 39 female prisoners. Interviews and questionnaire administration of 12point scale inventory. | Of the 11 married respondents, 72.7% felt the healthcare delivery in the female prison was poor. The majority of single women (53.6%) affirmed that there is poor accessibility to health care in the Kumasi female prisons.  Prison health caregiver was trained as a village health worker to provide health care service in places where there are no doctors.  The care giver indicated that while screening of inmates through physical examination was conducted prior to admission to rule out pregnancy, no laboratory examination was conducted.  No basic investigation equipment (functioning sphygmomanometer or thermometer) available.  Inmates who report with health problems are treated with available medication. If symptoms persisted for days or weeks, the inmate is sent to a hospital but there was a security risk.  Non-availability of drugs to cure common ailments like malaria, candidiasis, headache and fever observed  Pregnant inmates for ANC and with chronic conditions like ulcers or hypertension normally visit the hospital to see the doctor on duty.  Inmates with babies are retained and stayed until first birthday of the child after which social welfare or the family take custody of the child.  Depending on the type of sentence, they are restricted (handcuffed) while going for ANC or PNC (Security fears)  Inmates do frequently report with candidiasis, treated at the facility but some are not treated due to non-availability of drugs.  Reoccurrence of the disease (candidiasis) attributed to the single water closet available for all inmates.  Skin infections and bed bugs in the overcrowded cells.  Health service provider had not attended any refresher course or training.  Male inmates had a comparative advantage over female inmates as they were provided with everything including drugs and opportunities to update their skills. | Delivery of quality health service was hampered by lack of human resources as well as lack of basic diagnostic equipment and essential vital medicines. Without these key basic essentials in health service delivery, provision and accessibility to health care for women prisoners remains a challenge |
| Solomon OJ, Nwankwoala R | The plight of female prisoners in Nigeria and the dilemma of health rights violations. Soc Sci Pak. 2015; 10:5 1–7. | To discuss some of the main challenges faced by female inmates in terms of health and other social issues, and to suggest possible solutions. | Nigeria | Literature review | Irrespective of gender, correctional facilities described as having inhumane conditions, with over-crowded leading to deplorable health situations  Problems with provision of quality health care include inadequate health personnel, facilities and systems, lack of health care policies and standard operating procedures.  Most of female inmates are poor, educated and held in filthy prison environment, without basic sanitary products such as sanitary towels toiletries and soap.  An increase in the number of women inmates giving birth in prisons and yet not all prisons are equipped or prepared to handle growing baby population.  It is not uncommon for women in prison to discover that they are both pregnant and HIV positive.  Frequent sexual abuse and harassment from male wardens.  Most toiletries are provided by NGO and religious organizations  Female inmates rarely receive standard health care as most prisons do not have standard diagnostic equipment such as mammograms, CAT scan, MRI and pelvic examination  ANC rarely given  Women inmates engage in risky sexual behaviours that predispose them to STIs and HIV**.** | The inadequacy of and in accessibility of health facilities for female inmates compromises their health. |
| Nangia EN, Fontebo HN. | Treatment of Female Offenders in Prison: The Case of Cameroon.  Int J Humanit Soc Stud. 2017; 5:318–24. | To document and examine the treatment of deplorable prison conditions from female prisoners’ perspectives | Cameroon | Qualitative  Focus group discussion with 10 female inmates and employed a purposive sampling | Women offenders often not provided with sanitary towels in prison and during their menstruation they are forced to improvise.  Female prisoners plagued with problems related to pregnancy.  Differential attitude of prison staff towards female offenders  Poor diet  Toilets either bad and unfit for use or very unclean which caused inmates to use buckets.  No supply of water which exacerbate the situation.  Overcrowded due to limited space and obstructed ventilation  No toiletries like rolls, sanitary towels and pads  Health clinic only functional for pregnant inmates despite the presence of a health unit which is functional during the day.  Catholic church helping with medical expenses to access hospitals for ANC and delivery. | Skewed resource allocation within prisons resulted in Equity in access to services being affected by gender Additionally, unique reproductive health needs of female prisoners were not part of the package of health services which should be provided within prisons |
| van den Bergh BJ, Gatherer A, Møller LF | Women’s health in prison: urgent need for improvement in gender equity and social justice. Bull World Health Organ. 2009; 87:406–406. | To assess gaps in current arrangements for dealing with women offenders | Global | Editorial | Limited or no provision of HIV prophylaxis for prevention of mother-to-child transmission.  Limited access to highly active ART and basic primary healthcare  Women were noted to be at extreme risk of being beaten by other female inmates and of sexual abuse by police and prison officials. | Unavailability of HIV/AIDS interventions in prison settings continued to represent a concern. |
| Van den Bergh BJ, Gatherer A, Fraser A, Moller L. | Imprisonment and women’s health: concerns about gender sensitivity, human rights and public health. Bull World Health Organ. 2011; 89:689–694. | To report on imprisonment and women's health: concerns about gender sensitivity, human rights and public health. | Global | Literature scoping and policy analysis | Current provision of health care to imprisoned women fails to meet their needs and falls far short of what is required by human rights and international recommendations.  The evidence highlights a lack of gender sensitivity in policies and practices in prisons, violations of women’s human rights and failure to accept that imprisoned women have more and different health-care needs compared with male prisoners, often related to reproductive health issues, mental health problems, drug dependencies and histories of violence and abuse. | Prison health policy and practice not gender sensitive. |
| Agboola C. | Memories of the “inside”: Conditions in South African women’s prisons. SA Crime Q. 2016;19–26. | To examine the experiences of women prior to, during and after incarceration in South Africa. | South Africa | Qualitative Retrospective interviews with former women inmates in South African Prisons. In depth interviews with 10 ex-inmates | Overcrowding was reported with female inmates reportedly sleeping 4 to 6 people in one small room as like a cubicle, sometimes 2 sharing a bed sometimes inmates sleeping in corridors. Inadequate bathrooms  Complaints made by inmates regarding the alleged failure of correctional facilities to provide medical treatment.  Certain South African correctional facilities had poor and inadequate health care provision  Some institutions did not have doctors to attend to the medical needs of female prisoners.  A number of pregnant female inmates did not receive medical care at any point in their incarceration, and some pregnancies allegedly went unnoticed by the authorities.  Nurses were available to attend to inmates, however there were too few of them to meet their healthcare needs.  Wardens and nurses did not give proper attention to reports of ill-health from female inmates resulting in inmates resorting to treating their illnesses themselves, using home-made remedies. Prison food was monotonous, terrible, not properly cooked sometimes and did not constitute a balanced diet.  Some inmates could request special diets for health (diabetic and HIV-positive inmates) and religious (Halaal foods for Muslims) reasons, and that this food was generally of a better quality.  unhygienic and unsanitary conditions, which contributed to ill health  Water cuts in prisons and inadequate bathing soap | Health care, sanitation, food provision, access to education and reading materials, and, in particular, overcrowding are considerable challenges faced by the South African Department of Correctional Services |
| Sarkin J. | Prisons in Africa: an evaluation from a human rights perspective. Sur Rev Int Direitos Hum. 2008; 5:22–51. | To evaluate prisons in Africa from a Human Rights Perspective. | Africa | Literature review | Women prisoners are particularly vulnerable to sexual abuse by prison guards whether in female or mixed prisons.  The declaration merely calls for “particular attention” and “proper treatment” of women’s “special needs”.  Such vague aspirations—to say nothing of the wholesale omission of pregnant women—reflects a lack of political will and gender sensitivity in African prisons | Women’s access health in prisons is affected by lack of political will, policies and gender awareness, and is impeding progress towards improvement of women’s health |
| Modie-Moroka T, Sossou MA | Women, criminality and multifocal empowerment responses: some prospects for Botswana. J Soc Dev Afr. 2001;16. | To document the situation of women in prison in Botswana. | Botswana | Both qualitative and quantitative analysis of 80 life-history interviews with incarcerated women and girls in six prisons | Requirements could not be met for healthy lives, let alone for pregnant women, with inadequate nutrition, fresh air, recreational facilities, and sanitary conditions | Inadequacy of reproductive health services and empowerment programmes for women in prisons |
| Vetten L. | The imprisonment of women in Africa. Hum Rights Afr Prisons. 2008;134–154. | To assess human rights in African Prisons. | Several African Countries within SSA | Literature review | Women in African prisons mention ack of access to health care. Themes are;  Managing menstruation  Pregnancy and childcare,  Violence and abuse  Drawing together evidence on the lack of personnel qualiﬁed to provide mental health services  Lack of screening services for breast, ovarian and cervical cancer.  Untreated diseases, and high rates of mental illness, including attempted suicides. | Sexual and reproductive health services availability and access to health services in most SSA countries and is a challenge. This has implications on quality of life for prisoners and against fundamental basic human rights like the right to health of prisoners |
| Telisinghe L, Charalambous S, Topp SM, Herce ME, Hoffmann CJ, Barron P et al | HIV and tuberculosis in prisons in sub-Saharan Africa  www.thelancet.com Vol 388 September 17, 2016  1215-1227 | To investigate the epidemiology of HIV and tuberculosis in prison populations, describe services available and challenges to service delivery, and identify priority areas for programmatically relevant research in sub-Saharan African prisons. | 24 of 49 countries making SSA | Literature review and case studies in 5 SSA countries | Prison warden’s negative attitudes impacting on access to ARVs reported in by a female detainee in Rwanda  Female detainee in Uganda failing to access ARVs due to non-availability  Prison health care remains suboptimal.  All South African prisons have internal clinics but these clinics are understaﬀed.  A nursing act that prevents nurses prescribing without authorisation contribute to time lags and bottlenecks in chronic-disease management  Increased dependence on non-governmental organisations to deliver tuberculosis and HIV testing and treatment  Prevention services are weak because nurses frequently are not adequately trained in primary care or preventive health  Notable dearth of sex-disaggregated and age-disaggregated data describing HIV or tuberculosis treatment outcomes for women and children within sub-Saharan African prisons | Consistent supply and access to ARVs is key to maintaining optimal adherence to maximize on available less expensive therapies in low resourced SSA countries. A break in supply chain management of ARVs results in missed doses, suboptimal adherence and emergence of drug resistant strain of the virous beyond the prison cells. Disaggregation of data is key to inform targeted evidence-based interventions and resource allocation |
| Lorizzo T | Prison reforms in Mozambique fail to touch the ground: Assessing the experience of pre-trial detainees in Maputo. SA Crime Q. 2012; 2012:29–38. | To explore the conditions of detention and  access to legal representation of 20 pre-trial  detainees in the Central and Civil Prisons of  Maputo. | Mozambique | Qualitative utilizing private face-to-face interviews with the  detainees. The participants were selected by the  authorities of each prison. Open-ended  questionnaires. Numbers not provided | Prisons are characterised by old and degrading infrastructure. ‘As paredes estâo cansadas’ [The walls are tired],42 said one of the detainees interviewed in the Civil Prison. Although roofs were not leaking, walls are cracked.  Food is not distributed at regular times and the actual diet consists of a combination of porridge for breakfast, and rice, maize, beans or peanut sauce for lunch or dinner.  Female detainees in the Civil Prison said that they had sugar and hot water for breakfast.  In the female section of the Civil Prison there was one toilet, a sink and a shower; although one of the women interviewed said that there was a toilet in her cell.  One female detainee said that they used plastic bags to relieve themselves in the night.  In both institutions, a health ward opened from Monday to Friday, from 09h00 until 15h00. The service worked as a pharmacy rather than a health care centre  The doctor reportedly gave only Paracetamol for all the diseases you have. If one gets sick after three o’ clock in the afternoon one had to wait until the next morning and nothing is going to change because the only thing he is going to give is Paracetamol. A prisoner needs to pray God to not get sick from Friday until Monday  Transportation to the civil hospital of Maputo was rare and only happened when the detainee was grievously unwell, as the transfer of sick detainees is seen to increase the possibility of escape  Although access to health care in prison was restricted, this needs to be seen in context since 40% of the Mozambican population has no access to medical services. There are only three doctors and 21 nurses for every 100 000 people in Mozambique. There are approximately 600 doctors in the country. | Availability and accessibility of health services in prisons mirrored services available for the general population. However, the general population had an added advantage of being able to seek health services from other health providers unlike in prison settings. Supply chain management of medicines, including human and equipment resources should be addressed as a matter of urgency as per different protocols that the country has ratified. |
| Haffejee S, Vetten L, Greyling M | Exploring violence in the lives of women and girls incarcerated at three prisons in Gauteng Province, South Africa. Agenda. 2005; 19:40–47. | To explore and quantify violence in the lives of women and girls in conflict with the law. | South Africa | Quantitative cross-sectional interview survey with a sample of 348 women prisoners | Violence is not the only hardship more likely to be experienced by women in prison than non-imprisoned women  The conditions of women’s imprisonment have attracted almost no attention  11% of women said that they were currently involved in a consensual sexual relationship in prison while 5% said they had been coerced into a current sexual relationship.  1 in 3 women experienced physical violence in prison | Physical and sexual violence within prison settings is also a reality among female prisoners. Policies and interventions targeting both prison administrators and prisoners themselves need to be enforced and their implementation monitored |
| Alexander J | Death and disease in Zimbabwe’s prisons  Lancet  <https://www.ncbi.nlm.nih.gov/pubmed/19330896> | To describe the  conditions in two  Harare’s main prisons in late 2008, | Zimbabwe | Interviews with former prisoners and former and serving prison officers about the precarious conditions inside Zimbabwe's prisons. Numbers not provided | Describing the conditions in two of the capital city Harare's main prisons in late 2008, a prison officer explained that they had gone the whole year in which for prisoners and prison officer food was hand to mouth  Meals for prisoners were inadequate and getting at least one meal, a day was described as lucky sometimes they would go without even a meal  Due to malnutrition prisoners were described as moving skeletons, moving graves  Prisoners described how the sick and the healthy slept side by side, packed together like sardines,  Deaths from disease in Zimbabwe's prisons have risen since the start of the precipitous economic decline and political crisis that gripped Zimbabwe in the late 1990s. From 1998 to 2000, the Zimbabwe Prison Service estimated some 300 deaths per year due to disease, tuberculosis being the biggest killer.  The immediate causes of escalating prison deaths are not hard to find: severe overcrowding, broken, overflowing toilets, water and electricity cuts, a lack of blankets, uniforms, winter clothing, medicines and other commodities like soap, and severe food shortages  Antiretrovirals are rarely available, and the dietary requirements of treatment were not being met  Cholera outbreak in Harare's Central Prison killed on average four to five prisoners a day with a peak of 18 deaths in 1 day, according to prison officers. | High mortality and morbidity in prisons was exacerbated by inhuman and degrading conditions within the prison environment. Continental and international human rights organization should be the voice of the voiceless and interventions to remedy prison conditions should be designed from a rights -based perspective |
| **Evaluation Report** | | | | | | |
| Van Hout, MC., Mhlanga Gunda, R., Rusakaniko, S. | XSSV02 Independent project evaluation report 2017. [Internet]. [cited 2018 Jan 24]. Available from: https://www.unodc.org/documents/evaluation/Independent_Project_Evaluations/2017/XSSV02_independent_project_evaluation_report_2017.pdf | The goal was to conduct a Final Independent Evaluation of implementation of project XSS V02 fully in line with set UNODC Norms, Standards, Templates and Guidelines. | Angola, Ethiopia, Lesotho, Malawi, Mozambique, Namibia, Swaziland, Tanzania (+ Zanzibar), Zambia & Zimbabwe) | Qualitative document review, interviews with stakeholders (n=49) Core Learning partners prison wardens (n=64)  16 Focus group discussions with stakeholders (n=64) on line survey with prison wardens (n=6) | Women and their children are neglected in HIV Prevention Treatment Care and Support (PTC&S) programming,  A programming gap between incarceration and community continuum of HIV PTC&S, and target areas warranting specific initiatives for women, children, juveniles, Intravenous Drug Users (IDU), Man who have Sex with Man (MSM) and those affected by mental health conditions. | A comprehensive package of care for women prisoners and their children including other Most At-Risk Populations (MARPs) need to be urgently addressed. Specific needs of these populations should inform designing of interventions within prison settings based on identified gaps. |
| **Media Reports** | | | | | | |
|  | Assessment and treatment of female prisoners in Africa  [Internet]. [cited 2018 Jan 24]. Available from: http://www.unafei.or.jp/english/pdf/RS_No94/No94_VE_Agomoh1.pdf | To critically analyse how the African continent in which prisons rank low on various lists of priorities has failed to adequately address the plight of incarcerated women. | Several African countries including Nigeria but no list of the countries. provided. | Qualitative and quantitative. No numbers provided. | 9.3 % of the female prisoners interviewed were pregnant while 7.8 % delivered their babies while in prison.  Lack of adequate facilities for pregnant female prisoners and those with their babies in prison including pre-natal, post-natal and creche facilities, etc.  The shortage of water is usually more prevalent amongst prisons located in rural or semi-rural areas  74.4% reported that they receive medical attention in prison  25.6% reported that they did not receive any such care.  92% reported that medical examination and drugs were made available to the prisoners.  45.5 % reported that the female prisoners received ANC and PNC care in prison  54.5% reported that this was not provided  78.9 % reported that the babies with their mothers in prison enjoy medical attention in prison but 21.1% reported that this was not the case.  96.7 % of the respondents reported that they feed three times daily.  32.8 % complained about the quality of food they were given  No separate feeding arrangement is made for pregnant women and nursing mothers.  Pregnant and nursing mothers in prison feed on the same menu and budget as every other prisoner | Despite regional and international protocols that most governments have ratified within SSA, prison conditions instead of getting better seem to deteriorate more so for women prisoners |
|  | Newsday. Chikurubi Female Prison sitting on health bomb [Internet]. NewsDay Zimb. 2017 [cited 2018 Jan 26]. Available from: https://www.newsday.co.zw/2017/03/chikurubi-female-prison-sitting-health-bomb/ | To conduct a tour of the prison complex and explore perceptions of female inmates on their living conditions | Zimbabwe | Qualitative  Interview with prison Dr and female inmates at Chikurubi maximum women prison during a tour of the facility by Parliamentary Portfolio Committee on Women Affairs Numbers not provided | Inmates who got a chance to speak to the committee expressed concern at the deplorable conditions of prison cells, while others said they could not access medication for conditions such as diabetes, as there was a deliberate focus on HIV and AIDS.  Apart from the health challenges, inmates also complained about the poor food that was being served to them and appealed to donors to help in improving their diet.  “We do not have meat, sugar ran out in December and beans also ran out last week. Toilets sanitisers and buckets are scarce. Sinks are not working and there is no running water and pipes are down,” one of the inmates said.  No cot beds for children | Women in prisons face health challenges despite the country having ratified continental and international treaties on upholding human rights including that of prisoners and their health. |
|  | Zimbabwe: The untold stories of pregnant inmates [Internet]. Shout-Afr. 2015 [cited 2018 Jan 26]. Available from: http://www.shout-africa.com/news/zimbabwe-the-untold-stories-of-pregnant-inmates/ | To record experiences of ex-pregnant female prisoners at prisoners pregnant | Zimbabwe | Qualitative  Interviews with 3 ex-prisoners, Deputy Commissioner of prisons an NGO representative working with prisons and a church organization | The prison had almost 15 pregnant inmates at that time and officials had told them that the journey to delivery was going to be regrettable  Poor nutrition and overcrowding with 20 inmates sharing one large room  Informant claims that inhuman treatment prevailed in both the prisons and health institutions that they went to deliver their babies.  “…We had no regular medical check-ups. We were treated like ordinary female inmates. And then there was the abuse- They called us names…Some of the nurses shouted at me while in labour at Parirenyatwa Hospital where I had been taken in my prison uniform and handcuffs...”  A 2014 report on Zimbabwe Prison Conditions notes that, ‘prison conditions are harsh with incidences of poor levels of sanitation, overcrowding and inadequate medical facilities.  Another inmate shared the same story as she served jail time while pregnant:  “…The joys of motherhood are lost by the insane conditions that prevail in prisons because they are unsuitable for nursing and pregnant mothers...”  “…Pregnant inmates are treated just like any other female prisoner without their needs being recognised. The prisons do not have post- natal care… You are forced to return to jail within 48 hours after giving birth at public health facilities together with the newly born baby and that is when you get an extra blanket for the baby...”  “When my time to give birth was due, I was transferred to Harare Hospital. The prison garb tells its own story to both the health professionals and other expecting mothers who instantly stigmatize you. The nurses utter all sorts of abusive words.  “…It is depressing to give birth whilst you are in prison because of the conditions that we would be exposed to. The diet does not change for nursing mothers and many risk a lot of infections…”  Prisons deputy commissioner reported acute food shortages affecting the prison and nursing mothers were diagnosed as malnourished  Deputy commissioner said that prisons were saddled with multifaceted problems that were inhibiting it from providing basic needs for prisoners including food, water and clothing due to lack of inadequate funding from the fiscus  Toiletries used by inmates are provided by non-governmental and religious organizations.  Church working with the government and the Zimbabwe Prison Services holding awareness meetings to prison officials so that they change their attitude towards pregnant inmates and local hospitals also targeted to educate the health personnel on the rights of the pregnant inmates | Health professionals who have taken pledges such as the nurses’ pledge that guide their practice should not just do so in theory but walk the talk Awareness campaigns of needs of female prisoners and their treatment should be nationwide with interventions targeting different sub-groups of the nation should be designed and implemented. Inadequate resources should be addressed in a complementary way by both state and non-state actors |
|  | Spotlight: HIV Management in a Malian Women’s Prison [Internet]. TheBodyPRO.com. [cited 2018 Jan 26]. Available from: http://www.thebodypro.com/content/art34125.html | To highlight the issue of HIV among women in a Malian prison | Mali | Narrative describing HIV management in Malian Women ‘s Prison | Routine HIV testing does not exist in the women's prison.  Occasionally, HIV tests are performed when a woman is chronically ill, or goes to the hospital to have a child or undergo surgery.  As in Malian society, AIDS remains a taboo subject in the correctional environment.  Because of the stigma associated with AIDS in Mali, the women here simply do not believe the facts about AIDS," said Kouyaté. Not surprisingly, most incarcerated women here would likely refuse to be tested even if given the opportunity.  The administration has always made concerted efforts to keep testing as discreet as possible but personal information travels fast inside the prison walls.  These women are very perceptive," Kouyaté said. "They know what goes on here better than the staff does."  The Bollé prison does not have recourse to legislation on informed consent or any laws regarding HIV testing in the correctional setting. Upon further investigation of what is an extremely delicate -- but not a litigious -- issue in Malian corrections, Kouyaté acknowledged the need for protocols for a confidential and effective testing system.  Currently, the following ARVs are approved for treatment of HIV infection in Mali: didanoside, zidovudine, lamivudine, stavudine, nevirapine, efavirenz, nelfinavir and indinavir.  There are not enough of these medications, however, to keep them in stock at the pharmacy, which renders treatment interruption inevitable.  An infrastructure for dispensing these drugs does not exist, nor does the personnel for distributing them.  Furthermore, difficulties in accessing transportation to and from the hospital or clinic for treatment and proper follow-up are realities in Mali | Stigma remains the Achilles heel to HIV/AIDS uptake of interventions aimed at preventing further transmission of the virus. Delayed testing also affects access to early management of HIV as chronic disease leading to high mortality and morbidity among people living with the disease |
| **Thesis** | | | | | | |
| Makarati J | Breaking the silence of menstruation in Zimbabwe: Where does the female prisoner stand [PhD Thesis]. [cited 2018 Jan 18]. Available from  www.searcwl.ac.zw/index.php | To explore the way in which female prisoners have attempted to cope with their menstrual lives | Zimbabwe | Qualitative  Document review  Interviews with female prisoners (n=14)  Administrative Officer n=2)  Ex-Prison Officer (n=1)  Magistrate (n=1) | Women prisoners depended on prison officers for control of their menstruation  The one pad a day was a reality with some women claiming they received half a pad a day for use the whole day regardless of the flow  Disposal facilities were either insufficient or absent altogether  Essential underwear either limited or absent  One had first to mess themselves before being provided by another pad  Standard practice was that pads were distributed twice a day during medicine rounds with one pad being given during the morning round and another in the afternoon to be used at night  Prisoners reported that sometimes pads were cut into half so instead of getting a full pad one would get half a pad  Coping strategies to manage insufficient sanitary pads women would pull pieces of wool from the pad that is cut in half then roll it into small balls and insert them into the vagina like a tampon a practice they reported was painful  The female prisoners also used pieces of blankets, prison uniforms and woollen hats for babies in prison with their mothers  Unhygienic disposal of pads especially at night  Cells with no in built in toilets used 25 litre buckets as toilets | There is a glaring gap between statutory instruments and practice. Menstruation is a biological process that needs to be treated with dignity and respect for every woman regardless of status. The legislature and civic society should hold the government to account to ensure basic needs for women prisoners such as sanitary wear is adequately provided within prisons. |
| Twea S. | Women as offenders–the social and legal circumstances of women who commit crimes: A case study of selected prisons in Malawi. 2013. [cited 2018 Jan18] Available from  .ir.uz.ac.zw/handle/10646/1048 | To assess the social and legal implications of women who commit crime | Malawi | Qualitative  Interviews with 29 administrative staff at different levels and 30 women prisoners  Document review | One piece of soap allocated per week but currently two or three months can elapse without receiving any.  Prisoners eat once a day.  Children were being maintained on adult diets except in rare circumstances when soy flour could be provided for children.  Overcrowding in cells.  Cells are very narrow and make sleeping difficult.  Cells are poorly ventilated, dark and with low standards of hygiene.  Inmates reported that they are often sick from malaria, constipation and diarrhoea due to the poor food.  Some prisoners have serious health problems such as TB/HIV.  Although prisoners have access to the prison medical officer, routine medical check-ups for females are not carried out.  While medical care may be poor for all prisoners, the situation is far worse for women prisoners because prison health care systems were originally created for men.  Care is frequently only administered as an emergency.  There were also indications that children were not adequately accessing under five clinics and basic immunization against polio, TB, diphtheria and measles. | Living conditions within prison settings endangers the health of women prisoners and increase the transmission of communicable diseases need to be addressed as a matter of urgency. Overcrowding, health provision, inadequate manpower also affects availability and accessibility of health care  Poor nutrition and unbalanced, unhygienic conditions exacerbate transmission of communicable diseases. |
| Fontebo HN | Prison Conditions in Cameroon: the narratives of female inmates [PhD Thesis]. 2014. [cited 2018 Jan 24] Available from  uir.unisa.ac.za/bitstream/handle/10500/13069/thesis_fontebo_hn.pdf. | To help provide answers as to whether there is a discrepancy or variation between policy/laws and practice in relation to female inmates in prisons in Cameroon. | Cameroon | Qualitative  A total of 18 prison staff members and two NGO representatives  Open-ended narrative interviews with 18 female inmates Document review | Overcrowding, poor nutrition,  Lack of adequate training and specialisation of the prison staff.  Pregnant women do not receive adequate ante- and post-natal care in prison and their dietary requirements are normally not catered for.  Prisons described as ‘death chambers’ with the prisoners sleeping on dirty floors.  Communal cells are often poorly ventilated and badly lit, and lack adequate washing facilities.  Overflowing buckets in one corner of the cell usually serve as the only toilets.  Acute water shortages in some prisons have exacerbated the unsanitary conditions.  The poor conditions in these prisons often lead to severe health risks and a number of deaths from malnutrition, dehydration, dysentery and pneumonia.  The medical facilities are limited.  While female inmates need better healthcare than the male inmates, the prison authorities do not have the resources required to provide this.  The issue of hygiene and sanitation is related to that of medication.  In Kiri-Kiri prison female prisoners complained of having to use their hands and buckets to remove excreta from a full and overflowing suck-away and being exposed to stinking excreta in the yard  Female inmates in Cameroon, Uganda, Zimbabwe and Nigeria were not provided with either bathing soap or sponges even when soap was provided, it was once in a while in small quantities.  The prisoners bought these items with their own money, in order to avoid developing rashes.  The same applied to items such as sanitary towels and soap for washing clothes.  Those who are not able to afford such things, especially sanitary towels, resort to unsafe alternatives such as using discarded cloth or cutting off bedding foam, which is washed and re-used  In Zimbabwe, instead of sanitary towels, women used alternatives such as newspapers, tissues and pieces of blanket or prison uniform  The difficulties associated with menstruation were also compounded in Zimbabwe and Uganda | Common threads in the thesis similar to other studies include overcrowding, poor hygienic and sanitary conditions, inadequate ANC and PNC care. The intersecting factors affecting women’s health status warrant a holistic rights-based approach with a mix of stakeholders. |
| Kamau NJ | Access to health care by inmates in Kenya: a study of Lang’ ata women’s prison and Nairobi remand/allocation [Internet] [Thesis]. 2006 [cited 2018 Jan 24]. Available from: http://erepository.uonbi.ac.ke:8080/xmlui/handle/11295/17516 | To assess access to health care by inmates in Kenya: A study of Lang’ata women ‘s prison and Nairobi Remand Allocation | Kenya | Quantitative and qualitative  The questionnaires were administered to 30 males and 29 females  inmates and 8 key informants | There exists an elaborate system within which inmates’ access treatment while they are in prison or custody.  Barriers to access to medical care included bureaucracy and negative attitudes by medical personnel and prison warders.  Delays, long queues and pathetic conditions.  Lack of resources essential medicines, medical personnel and facilities  Female prisoners able to obtain essential medicines and treated by doctors and nurses.  Female prisoners also managed to get referrals to better health facilities, which had x-ray and laboratory facilities such as Kenyatta National Hospital  Majority of the inmates complained about the poor diet and its preparation, which they said led to their poor health and stomach problems.  It was the view of the voluntary health worker that the prisons lack water resulting to poor sanitary conditions. | Access to medical care, qualified personal and medicines was better for female in mates compared to their male counter parts. |
| **United Nations Joint Programme on HIV/AIDS ( UNAIDS) United Nations Children's Fund (UNICEF ), United Nations Office of Drugs and Crime (UNODC), United Nations Political Office for Somalia (UNPOS) and Human Rights reports** | | | | | | |
|  | Assessment of the Prison System in Mogadishu South 2012. [Internet]. [cited 2018 Feb 8]. Available from: https://unpos.unmissions.org/unpos-and-human-rights | To assess the prisons sector in South Central Somalia/Mogadishu. | Central Mogadishu | Inspection and qualitative interviews  Series of visits to the Mogadishu Central Prison and meetings with Government officials from various institutions within the criminal justice sector, including representatives from the Ministry of Justice, Religious Affairs and Endowment, Custodial Corps, and the Judiciary. Consultations with representatives of the Civil Society and interviews with prisoners detained at the Mogadishu Central Prison. | Children in prison with their mothers  Water shortage and no running water in the female wing  Medical care lacking  Shortage of equipment and staff  No soap | Availability of health services for prisoners should be equivalent to that of the general population general public |
|  | Chad: Prisoners’ lives are threatened by appalling detention conditions \| Amnesty International [Internet]. [cited 2018 Jan 27]. Available from: https://www.amnesty.org/en/press-releases/2012/09/chad-prisoners-lives-are-threatened-appalling-detention-conditions-2012-09/ | To monitor human rights compliance | Chad | Report | Prisoners not medically checked on arrival.  No medical services available nor medical doctor permanently attached to any prisons at the end of June 2012.  In some cases, a nurse was appointed to a prison on a temporary basis. Work of the nurse hindered by the lack of medicines and basic medical facilities.  There was no space provided for a clinic in many prisons, such as Sahr and Amsinene prison in N’Djamena.  Rooms allocated as on-site clinics in some prisons were often empty or used for other purposes.  In other prisons, the clinic spaces were transformed into cells because of overcrowding.  No dedicated medical prison personnel in the Chadian prison system and the prison law provisions are not clear on this issue.  In some cases, the National Gendarmerie appoints its own members to serve as nurses or health personnel in a few prisons, decisions which are often made without consultation with the Ministry of Health and Ministry of Justice.  The lack of basic facilities and essential drugs complicated the work of the appointed nurses or health personnel.  No health workers or clinic at the newly opened prison.  Prisoners who were seriously ill were taken to N’Djamena general hospital, where family members have to buy medicine and pay the hospital bills.  Prisoners asked by officials to provide medical assistance to sick inmates without verification or assessment of the inmate’s medical training or skills. | The physical environment health provision and accessibility within SSA are all affected by intersecting factors and challenges that need a multifaceted response from the Justice system and other stakeholders.  Though the report does not specifically address women’s health needs, some findings are general and therefore affect both sexes |
|  | The HIV and TB Prison Crisis in Southern Africa \| Human Rights Watch [Internet]. [cited 2018 Feb 8]. Available from: https://www.hrw.org/news/2010/07/23/hiv-and-tb-prison-crisis-southern-africa | To highlight the double epidemic of HIV/AIDs and TB | South Africa | Editorial  General description of poor prison conditions in SA and Zambia and how they contribute to the double epidemic | Overcrowding  Lack of food and soap  Prisons are poorly resourced  Lack of financial resources | SSA has a double epidemic in terms of HIV/AIDS and TB prevalence hence the urgent need or measures to curb the spread of both epidemics in SSA prisons. The need to aggressively address the 2 epidemics is in line with the observation prison health is good public health practice |
|  | HIV, TB, and Abuse in Zambian Prisons \| HRW [Internet]. [cited 2018 Feb 8]. Available from: https://www.hrw.org/report/2010/04/27/unjust-and-unhealthy/hiv-tb-and-abuse-zambian-prisons | To analyse prison health conditions in Zambia by independent human rights organizations. | Zambia | Qualitative interviews with 246 prisoners, eight former prisoners, 30 prison officers  Facility tours | Overcrowding  Unhygienic conditions  Water is unclean or unavailable;  Soap not provided by the government.  Food insufficient and nutritionally inadequate, and has become a commodity traded for sex or labour in the prisons.  Limited access to ANC and PMTCT.  Inadequate toilet and bathing facilities | Conditions of incarceration in Zambian prisons exacerbate transmission of communicable diseases. The limited access to ANC and PMTCT services predisposes women to pregnancy induced conditions and exposes unborn babies to vertical transmission of HIV. |
| **African Union Reports** | | | | | | |
|  | Malawi: Mission on Prisons and Conditions of Detention, 2001 / Malawi / States / ACHPR [Internet]. [cited 2018 Jan 26]. Available from: http://www.achpr.org/states/malawi/missions/prisons-2001/ | To assess and document the conditions of detention in Malawi. | Malawi | Consultations, inspections and interviews 13 prisons visited, 4 consultations with authorities, 6 with NGOs, 5 with media houses and prisoners’ interviews in private, individually or in groups in each prison and police station visited. | Overcrowding and the lack of space  Sewerage system in bad state because of lack of maintenance and the high numbers of prisone.rs  Overcrowding leads to very poor hygiene.  Toilets and shower cubicles are not sufficient and no access to toilets at night.  There were no cleaning tools, nor disinfectant used in the prisons visited.  The provision of soap is poor.  Quantity and quality of food both inadequate and poor.  Inadequate health facilities.  Lack of proper management, medicines, medical staff and basic tools.  Lack of transport with long delays for transfer to hospital  Very long delay usually taken by prison authorities in finding a vehicle for referred sick prisoners.  Lack of proper attention and care in prisons where there is no health facility  Rape | Multifaceted approach needed to improve prison conditions as allocation of resources from the fiscus is inadequate to address needs of prisons and prisoners |
|  | Namibia: Mission on Prisons and Conditions of Detention - 2001 / Namibia / States / ACHPR [Internet]. [cited 2018 Jan 26]. Available from: http://www.achpr.org/states/namibia/missions/prisons-2001/ | To assess and document the conditions of detention in Namibia | Namibia | Interviews, Observations and document review of 10 prisons  Consultations with policy makers at various levels and administrators, various stakeholders including NGOs and the media, individual prisoners and of in groups | Interruption of water supply in some prisons.  Faulty facilities causing major unhygienic conditions (stagnant dirty water, smells, waste)  No cleaning materials  Overcrowding in police cells  Lack of medical staff  Delay in transferring sick prisoners to hospitals and specialised centres due to lack of a vehicle insufficient staff, fear of prisoners' escaping, and lack of privacy for females during consultations | Provision of health services is dependent on availability of adequate staffing levels for both medical and paramedical professionals including all key resources for ancillary services to ensure provision of quality services in line with recommended service standards |
|  | Uganda: Mission on Prisons and Conditions of Detention - 2001 / Uganda / States / ACHPR [Internet]. [cited 2018 Jan 26]. Available from: http://www.achpr.org/states/uganda/missions/prisons-2001/ | To evaluate and document conditions of detention in Uganda | Uganda | Interviews, meetings Observations and document review  13 prisons visited, 6 police stations, 1 remand home for juveniles, discussion conducted with 7 authorities3 NGOs representatives, 9 representatives of media houses and individual prisoners and of in groups | No access to water and had to collect it from the kitchen  Mbale women prison was dilapidated.  Ceilings let water infiltration and there was no light. Windows had bars only.  The lack of variety in food.  In several prisons, they said they received no pads, no soap, no detergents or toilet paper.  Lack of trained personnel.  Lack of transport for transporting prisoners.  Lack of consulting rooms. | Basic amenities lacking affecting access to health care and service provision. The Justice system cannot address all these challenges on its own but need a coordinated approach that includes the private sector, local and international NGOs |
|  | Mozambique: Mission on Prisons and Conditions of Detention, 2001 / Mozambique / States / ACHPR [Internet]. [cited 2018 Jan 26]. Available from: http://www.achpr.org/states/mozambique/missions/prisons-2001/ | To evaluate and document conditions of detention in Cameroon. | Cameroon | Interviews, meetings observations and document review  8 prisons and 4 police stations visited. Consultations with 24 with authorities, 4 NGOs, 1research institutions and 8 media houses | Lack of basic amenities like soaps and toiletries, and the alarming rate of congestion.  There is a strong stench in the various premises. Police cells emit the worst odour (hygiene and toilets)  Sanitation materials are not regularly and adequately issued to inmates.  Water is highly scarce and there is a deplorable waste management.  The menu is poor and the quantity inadequate.  Food badly cooked, insufficient, and lacking good taste.  Lack of medical staff.  Delay in transferring sick prisoners to hospitals and specialised centres due to lack of transport.  Delays in transferring inmates to hospital prison authorities.  Severely ill inmates may have to wait several days before they could be taken to the hospital. | Conditions impacting on health service provision to women prisoners are similar to other mission reports and need a holistic approach to addressing them |
|  | Ethiopia: Mission on Prisons and Conditions of Detention, 2004 / Ethiopia / States / ACHPR [Internet]. [cited 2018 Jan 26]. Available from: http://www.achpr.org/states/ethiopia/missions/prisons-2004/ | to monitor prisons and other places of detention in Member States of the African Union | Ethiopia | Qualitative meetings, observations and document review  visited and inspected nine prisons, two prison farms and two police stations | Less overcrowded compared to men.  Few medical facilities.  Shortage of water and dirty toilets.  Expectant mothers, nursing mothers, children and sick prisoners are given special meals.  Women are not provided with, among other things, sanitary pads. In the Addis Ababa prison, the ICRC is providing women with some cloth which they use and wash to reuse.  Other basic items such as soap, sheets, and detergents not provided. | Despite having a shortage of health facilities and other necessities the provision of nutrition to nursing mothers and those who are sick and children ensures adequate nutrition provision for the prisoners. One country provided a special diet for pregnant, nursing mothers |
|  | South Africa: Prisons and Detention Conditions, 2004 / South Africa / States / ACHPR [Internet]. [cited 2018 Jan 26]. Available from: http://www.achpr.org/states/south-africa/missions/prisons-2004/ | To draw the attention of prison officials to the numerous lapses in the criminal justice system in general and the treatment of persons deprived on their liberty in particular | South Africa | Qualitative and quantitative Inspections, interviews, meetings with national and local prison officials, closed door meetings with detainees, and communications from civil society organisations and information from various organisations i.e. 19 Detention centres, 1 mental hospital, 1, repatriation centres, 6 prisons, Child Justice Centre 1, Prisons 6, Police stations 4, youth centre 1 and 8 institutions | Overcrowding due to high numbers of detainees.  Mother child Unit in Durban and the children are provided with special diet.  All the prisons visited, had an in-house clinic that provides first aid treatment for minor illnesses  In house clinics lacked l laboratory equipment or have very few medicines in the dispensaries (lack of medication).  Accessibility to outside hospitals was affected by reluctance of the authorities to take them to hospital, as women prisoners are only taken to hospital when their health conditions have seriously deteriorated.  Provision of basic items like soap, and detergents for washing clothes. | Failure to quickly process detainees and conclude cases overstretches resources. Despite these findings it was observed that in prison children were provided with meals and primary health care clinics available. within prisons unlike in most SSA countries one |
|  | Mozambique: Mission on Prisons and Conditions of Detention, 2001 / Mozambique / States / ACHPR [Internet]. [cited 2018 Jan 26]. Available from: http://www.achpr.org/states/mozambique/missions/prisons-2001/ | To assess the implementation of the recommendations made by the Report of the Special Rapporteur in 1997 | Mozambique | Inspections, interviews, meetings observations and document review. 8 prisons visited and 1 police station, Consultations undertaken with prison authorities 16 including contracted physicians, nurses and helpers at places visited, NGOs 8, and prisoners | Overcrowding  Inadequate health centres and medical staff  Food and water/sanitation problems.  Hygiene problems due to lack of soap, of cleaning tools, and limited access to bathing face ties and water.  No transport to ferry sick people to hospital | Intersecting factors impact negatively on the welfare and health of prisoners are observed. Given multiplicity of factors a coordinated approach to service availability and accessibility by different partners is warranted. |
|  | 52nd Ordinary Session / ACHPR [Internet]. [cited 2018 Jan 24]. Available from: http://www.achpr.org/sessions/52nd/ | To assess progress made addressing and implementing recommendations made to different countries taken by the mission and also challenges impacting on implementation | All countries in Africa and including SSA | Presentations, meetings and plenary discussions | With regard to women, the Protocol to the African Charter on the Right of women in Africa guarantees the right to integrity and security of women, pregnant and nursing mothers in detention, prohibits sexual violence in private and public and imposition of death penalty on pregnant and nursing mothers  There are several critical problems faced by women in prison - most are unmet in the prison environment. Women in prison have experienced victimization, unstable family life, school and work failure, and substance abuse and mental health problems.  Women prisoners are particularly vulnerable to sexual abuse by prison guards whether in female or mixed prisons  Prisons are not a safe place for pregnant women, babies and young children and it is not advisable to separate babies and young children from their mothers.  Lack of sufficient treatment facilities is increasing the risk of mother-to-child HIV transmission. | Despite statutes being in place and most governments ratifying these declarations and attempting to utilize a rights-based approach to interventions conditions in prisons in Africa including SSA remain poor with women and children mostly affected |
